# CTLA‐4 and HLA‐DQ are key molecules in the regulation of mDC‐mediated cellular immunity by Tregs in severe aplastic anemia

**DOI:** 10.1002/jcla.23443

**Published:** 2020-07-03

**Authors:** Bingnan Liu, Yuanyuan Shao, Xiaowei Liang, Dan Lu, Li Yan, Alexey Churov, Rong Fu

**Affiliations:** ^1^ Department of Hematology Tianjin Medical University General Hospital Tianjin PR China

**Keywords:** cytotoxic T lymphocyte antigen 4, human leukocyte antigen, regulatory T cells, severe aplastic anemia

## Abstract

**Background:**

Regulatory T cells (Tregs) inhibit the activation of cluster of differentiation (CD) 4^+^, CD8^+^T cells and the antigen‐presenting process of antigen‐presenting cells, and may play an important role in acquired severe aplastic anemia (SAA).

**Methods:**

Flow cytometry was used to measure CD4^+^CD25^+^CD127^dim^ Tregs, cytotoxic T lymphocyte antigen 4 (CTLA‐4) expression on Tregs, and human leukocyte antigen (HLA)‐DQ expression on myeloid dendritic cells (mDCs). The correlations of CTLA‐4 and HLA‐DQ with immune status and clinical indicators and the changes in these indicators after immunosuppressive therapy (IST) were analyzed.

**Results:**

In SAA patients, the number of Tregs and their CTLA‐4 expression were low but recovered after IST; the HLA‐DQ expression on mDCs was high but decreased after IST. The CTLA‐4 expression on Tregs and the HLA‐DQ expression on mDCs showed a negative correlation. The CTLA‐4 on Tregs was positively but HLA‐DQ on mDCs negatively correlated with the number of Tregs, natural killer (NK) cell number, and CD4^+^T/CD8^+^T ratio. CTLA‐4 was positively but HLA‐DQ negatively correlated with the percentage of granulocytoid and erythroid cells in bone marrow, white blood cell count in PB, absolute neutrophil count in PB, and the percentage of reticulocytes in PB.

**Conclusions:**

CTLA‐4/HLA‐DQ may be key in the regulation of Tregs on mDCs in SAA patients. Our findings should be helpful for further investigation of the mechanism of immune pathogenesis in SAA patients. Studies on the regulators of Treg and CTLA‐4 activity will be valuable for SAA therapeutic target research and disease monitoring.

## Graphical abstract

CTLA‐4 and HLA‐DQ regulate cellular immunity by Tregs and mDC in SAA. A, CD4^+^CD125^+^CD127^dim^/PBL in the SAA, RSAA, and HC groups. B, CTLA‐4^+^Treg/Treg in the SAA, RSAA, and HC groups. C, HLA‐DQ^+^mDC/mDC in the SAA, RSAA, and HC groups. D, Negative correlation between the CTLA‐4 expression on Tregs and the HLA‐DQ expression on mDCs in SAA patients. ***P < .001, **P < .01, *P < .05. HC, healthy control (n = 24); RSAA, recovering severe aplastic anemia (n = 23); SAA, severe aplastic anemia (n = 21).

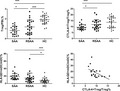

## 1 INTRODUCTION

Acquired severe aplastic anemia (SAA) is a bone marrow failure disease caused by abnormal cellular immunity. Its immune pathogenesis is that under the stimulation of unknown antigens, myeloid dendritic cells (mDCs) become hyperfunctional, the ratio of T‐helper 1 (Th1) and Th2 cells is out of balance, and the abnormally activated cluster of differentiation (CD)8^+^ T cells become hyperfunctional, attacking the bone marrow hematopoietic cells, which causes severe pancytopenia. Disorders of autoimmune tolerance also play an important role in the pathogenesis of SAA.^1^, ^2^

Regulatory T cells (Tregs) were first proposed to exist by Sakaguchi in 1995. Tregs inhibit the activation of CD4^+^ and CD8^+^ T cells, inhibit the antigen‐presenting process of antigen‐presenting cells (APCs), are a kind of T‐cell subset with immunomodulatory function, and play an important role in maintaining autoimmune tolerance. Tregs express many functional molecules, such as CD25 (interleukin [IL]‐2Ra chain), forkhead box protein 3 (FoxP3), and cytotoxic T lymphocyte antigen (CTLA‐4, CD152). FoxP3 is an important protein necessary for Tregs to exert their inhibitory function.^3^ CD4^+^ T cells expressing FoxP3^+^ with a high level of CD25 and low level of CD127 are referred to as having the phenotype FoxP3^+^CD25^high^CD127^low^CD4^+^.^4^ The CD127 expression is closely related to the regulatory function of Tregs and is negatively correlated with the FoxP3 expression and the inhibitory activity of Tregs.^5^ As our understanding of Tregs has grown, an increasing number of researchers have used CD4^+^CD25^+^CD127^dim^ as the molecular marker of Tregs. Treg dysfunction is associated with several common autoimmune diseases, including SAA.^6^, ^7^ In this study, we performed correlation analysis on the expression levels of CTLA‐4 and human leukocyte antigen (HLA)‐DQ on the surfaces of Tregs and mDCs, respectively, in the peripheral blood (PB) of SAA patients and explored the possible mechanism by which Tregs regulate mDCs in the SAA immune pathogenesis.

## 2 OBJECTS AND METHODS

### 2.1 Study subjects

Twenty‐one SAA patients admitted to the Tianjin Medical University General Hospital between January 2018 and December 2019 were enrolled (SAA group), including 13 males and 8 females, with a median age of 38 (9‐77) years. Twenty‐four recovering SAA patients were enrolled (RSAA group), including 11 males and 12 females, with a median age of 34 (12‐73) years. Twenty‐four healthy controls (HC group) were also enrolled, including 12 males and 12 females, with a median age of 33 (18‐66) years. The diagnosis of SAA was based on the standards of the International AA Working Group,^8^ and patients with paroxysmal nocturnal hemoglobinuria, myelodysplastic syndrome, cancer, infection, or congenital diseases were excluded. The patients in the RSAA group were all diagnosed and treated in our hospital. After effective enhanced immunosuppressive therapy (IST) with rabbit anti‐human thymocyte immunoglobulin (rATG) and cyclosporine (CsA), patients all had improved hematopoietic parameters, no dependence on transfusion of blood components, and reduced CsA usage.^9^ A total of 10 SAA patients diagnosed in our hospital, whose enhanced IST with rATG and CsA was effective, were selected to study the changes in immune status and clinical indicators before and after IST. The HC group included healthy volunteers. The clinical characteristics of the study subjects are shown in Table 1. This study was approved by the hospital ethics committee, and all study subjects signed informed consent forms.

**TABLE 1:** Clinical characteristics of SAA patients and HCs

	SAA (n = 21)	RSAA (n = 23)	HC (n = 24)
Male/female	13/8	11/12	12/12
Age (y)	38 (9‐77)	34 (12‐73)	33 (18‐66)
Bone marrow karyotype	Male 46,XY [20], female 46,XX [20]	Male 46,XY [20], female 46,XX [20]	Male 46,XY [20], female 46,XX [20]
Treatment	Untreated	IST (ATG + CsA)	No
WBC (×10^9^/L)	2.25 ± 0.93	6.96 ± 3.55	7.50 ± 1.93
ANC (×10^9^/L)	0.52 ± 0.45	2.25 ± 0.93	4.67 ± 1.14
ALC (×10^9^/L)	1.42 ± 0.56	3.14 ± 2.07	3.90 ± 1.86
HGB (×10^9^/L)	84.39 ± 43.34	95.29 ± 43.71	136.84 ± 17.49
PLT (×10^9^/L)	21.41 ± 12.79	71.57 ± 55.18	209.52 ± 42.55
Ret (ⅹ10^9^/L)	22.56 ± 19.63	85.73 ± 46.28	68.13 ± 61.08
Ret (%)	1.03 ± 0.82	2.42 ± 1.29	1.79 ± 2.44
CD4^+^T/CD8^+^T	0.55 ± 0.22	1.01 ± 0.38	1.62 ± 0.39
CD8^+^T/PBL (%)	52.70 ± 18.15	33.60 ± 10.86	23.59 ± 6.96
NK/PBL (%)	10.45 ± 4.87	14.75 ± 5.83	19.79 ± 4.93
Percentage of granulocytoid and erythroid cells in bone marrow (%)	16.95 ± 13.29	71.50 ± 17.57	84.00 ± 12.05
Percentage of lymphoid cells in bone marrow (%)	83.05 ± 13.29	27.50 ± 17.43	14.00 ± 11.86
Megakaryocyte number in bone marrow	1.29 ± 2.87	3.04 ± 5.05	19.21 ± 42.54

Note

Values are expressed as mean ± SD. Age values are expressed as median (min‐max).

Abbreviations: ALC, absolute lymphocyte count; ANC, absolute neutrophil count; HC, healthy control; HGB, hemoglobin; NK, natural killer cells; PBL, peripheral blood lymphocyte; PLT, platelet count; Ret, reticulocyte; RSAA, recovering SAA; SAA, severe aplastic anemia; WBC, white blood cells.

### 2.2 Main reagents and instruments

Mouse anti‐human monoclonal antibodies against CD4‐PerCP, CD25‐FITC, CD127‐APC, CTLA‐4‐PE, CD11c‐PerCP Cy5.5, CD123‐APC 750, Lin‐APC, HLA‐DR‐FITC, and HLA‐DQ‐PE and mouse IgG1‐PE were purchased from BD Pharmingen and BECKMAN COULTER Inc. Red blood cell lysis buffer was purchased from BD Biosciences. The flow cytometer models were FACS‐Calibur (BD Biosciences) and CytoFLEX (BECKMAN COULTER). Analysis software FlowJo 7.6.1 (TreeStar) and CytExpert (BECKMAN COULTER) were used.

### 2.3 Experimental methods

Flow cytometry was used to detect the HLA‐DQ expression on Tregs and CTLA‐4 expression on mDCs in PB. Fresh blood samples collected in the morning were put in ethylenediaminetetraacetic acid (EDTA) for anticoagulation. After anticoagulation, the blood samples (100 μL/tube) were thoroughly mixed with specific fluorescently labeled monoclonal antibodies (mAbs) and stained for 45 minutes. Then, 2 mL of red blood cell lysis buffer was added, and the tube was incubated for 10 minutes in the dark to remove red blood cells. After centrifugation at 1300 rpm for 5 minutes, the supernatant was discarded. After washing with phosphate‐buffered saline, the cells were resuspended in 300 μL for detection, and at least 50 000 cells/tube were recorded. In this study, CD4^+^CD125^+^CD127^dim^ defined Tregs, HLA‐DR^+^CD11c^+^Lin^‐^CD123^‐^ defined mDCs, and the mouse IgG1 was used as the isotype control to analyze the expression levels of CTLA‐4 and HLA‐DQ.

### 2.4 Statistical analysis

SPSS 19.0 software (IBM) was used for statistical analysis. The Kolmogorov‐Smirnov test was used to determine whether the data were normally distributed. When the data were normally distributed, the independent‐sample *t* test was used to compare values between groups. When data were not normally distributed, Kruskal‐Wallis one‐way analysis of variance (ANOVA) was used to compare values between groups. Correlation analysis between variables was performed using the Spearman rank correlation test. When a significant result was found, the correlation coefficient (*r*) is reported. A paired‐sample *t* test was used to analyze the changes from before to after the IST. Quantitative data are expressed as the mean ± SD, and *P* < .05 was considered statistically significant.

## 3 RESULTS

### 3.1 The number of Tregs and CTLA‐4 expression in SAA patients and HCs

**FIGURE 1:**
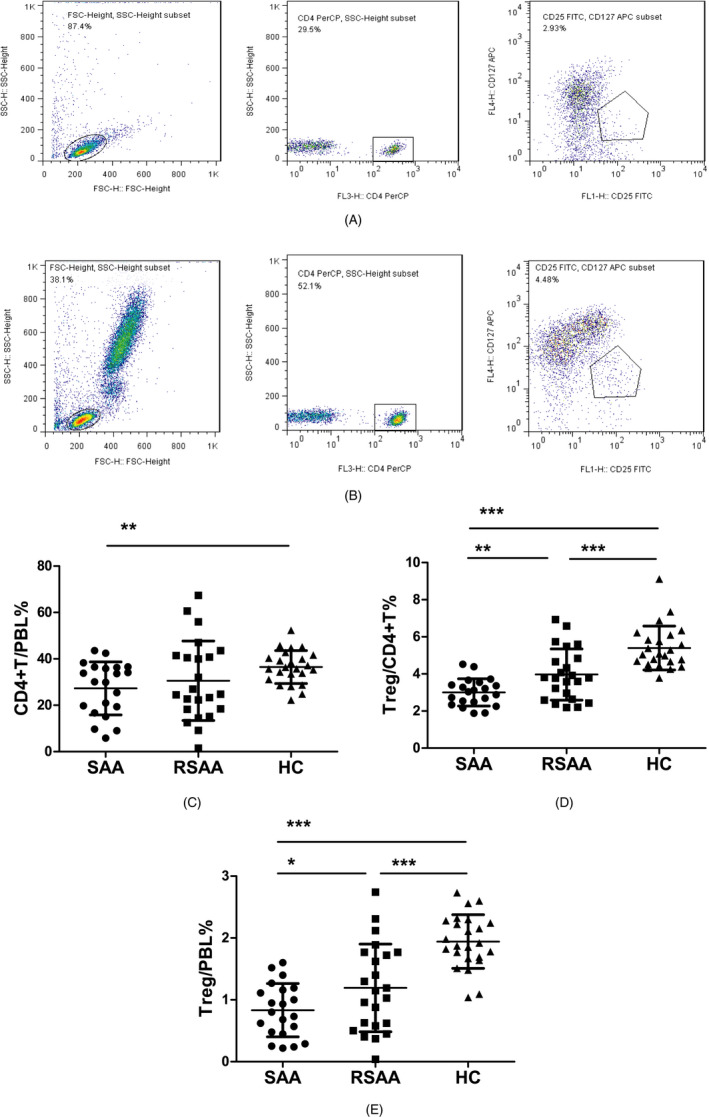
The number of Tregs in PB of SAA patients and HCs. A, Flow cytometry analysis of CD4^+^CD125^+^CD127^dim^ cells in PB of the SAA group. B, Flow cytometry analysis of CD4^+^CD125^+^CD127^dim^ cells in PB of the HC group. C, CD4^+^T/PBL in the SAA, RSAA, and HC groups. D, CD4^+^CD125^+^CD127^dim^/CD4^+^T in the SAA, RSAA, and HC groups. E, CD4^+^CD125^+^CD127^dim^/PBL in the SAA, RSAA, and HC groups. ***P < .001, **P < .01, *P < .05. HC, healthy control (n = 24); RSAA, recovering severe aplastic anemia (n = 23); SAA, severe aplastic anemia (n = 21)

**FIGURE 2:**
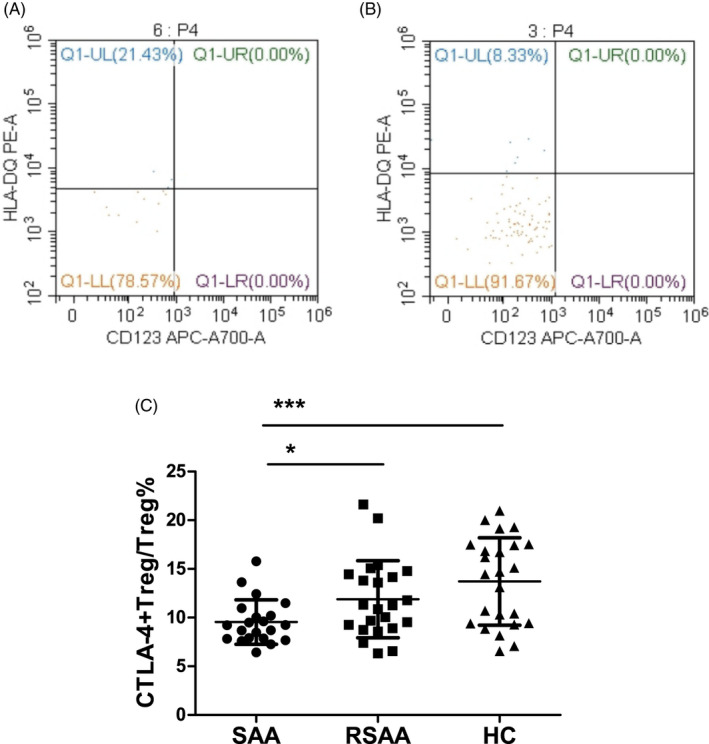
CTLA‐4 expression on Tregs in PB of SAA patients and HCs. A, Flow cytometry analysis of CTLA‐4 expression in CD4^+^CD125^+^CD127^dim^ cells in PB in the SAA group. B, Flow cytometry analysis of CTLA‐4 expression in CD4^+^CD125^+^CD127^dim^ cells in PB in the HC group. C, CTLA‐4^+^Treg/Treg in the SAA, RSAA, and HC groups. ***P < .001, *P < .05. HC, healthy control (n = 24); RSAA, recovering severe aplastic anemia (n = 23); SAA, severe aplastic anemia (n = 21)

The ratio of CD4^+^ T cells among the PBLs (CD4^+^T/PBL) in the SAA, RSAA, and HC groups was 27.28 ± 11.43%, 30.57 ± 17.12%, and 36.47 ± 7.13%, respectively (Figure 1A,B). The CD4^+^T/PBL in SAA group was significantly lower than in the HC group (*P* < .01), and there was no significant difference between the RSAA and HC groups (Figure 1C). In the SAA group, the percentage of Tregs in CD4^+^ T cells (Treg/CD4^+^T) was 3.01 ± 0.73%, which was significantly lower than that (3.97 ± 1.38%) in the RSAA group (*P* < .01) and that (5.39 ± 1.18%) in HC group (*P* < .001) (Figure 1A,B,D), and the Treg/CD4^+^T ratio in the RSAA group was significantly lower than that in the HC group (*P* < .001, Figure 1D). The product of Treg/CD4^+^T and CD4^+^T/PBL equals the ratio of Tregs among PBLs (Treg/PBL). The Treg/PBL ratio in the SAA group was 0.83 ± 0.43%, significantly lower than that (1.19 ± 0.71%) in the RSAA group (*P* < .05) and that (1.94 ± 0.43%) in HC group (*P* < .001), and the Treg/PBL ratio in the RSAA group was significantly lower than that in the HC group (*P* < .001, Figure 1E). The expression percentage of CTLA‐4 on Tregs in the PB in the SAA group (CTLA‐4^+^Treg/Treg) was 9.55 ± 2.28%, which was significantly lower than that (11.88 ± 3.96%) in the RSAA group (*P* < .05) and that (13.71 ± 4.48%) in the HC group (*P* < .05). There was no statistically significant difference in CTLA‐4^+^Treg/Treg between the SAA and HC groups (*P* < .001) (Figure 2A‐C).

### 3.2 HLA‐DQ expression on mDCs in SAA patients and HCs

**FIGURE 3:**
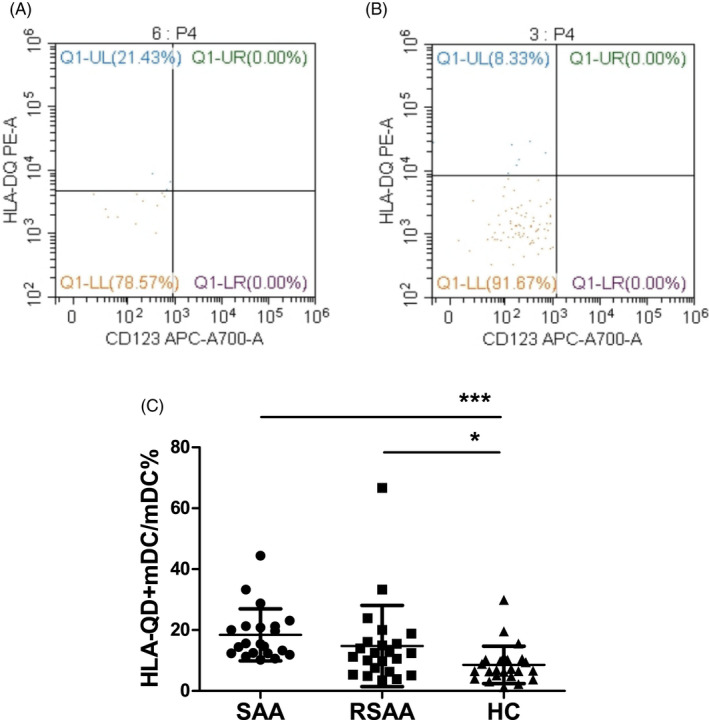
The expression levels of HLA‐DQ on mDCs and CD40 in PB in SAA patients and HCs. A, Flow cytometry analysis of HLA‐DQ expression on mDCs in PB in the HC group. B, Flow cytometry analysis of HLA‐DQ expression on mDCs in PB in the HC group. C, HLA‐DQ^+^mDC/mDC in the SAA, RSAA, and HC groups. ***P < .001, *P < .05. HC, healthy control (n = 24); RSAA, recovering severe aplastic anemia (n = 23); SAA, severe aplastic anemia (n = 21)

The expression percentage of HLA‐DQ on mDCs (HLA‐DQ^+^mDC/mDC) from PB in the SAA and RSAA groups were 18.44 ± 8.53% and 14.88 ± 13.33%, respectively, significantly higher than that (8.59 ± 6.14%) in the HC group (*P* < .001, *P* < .05). The HLA‐DQ^+^mDC/mDC in the SAA group was not significantly different from that in the RSAA group (Figure 3).

### 3.3 Correlation analysis of CTLA‐4 expression on Tregs and HLA‐DQ expression on mDCs in SAA patients

**FIGURE 4:**
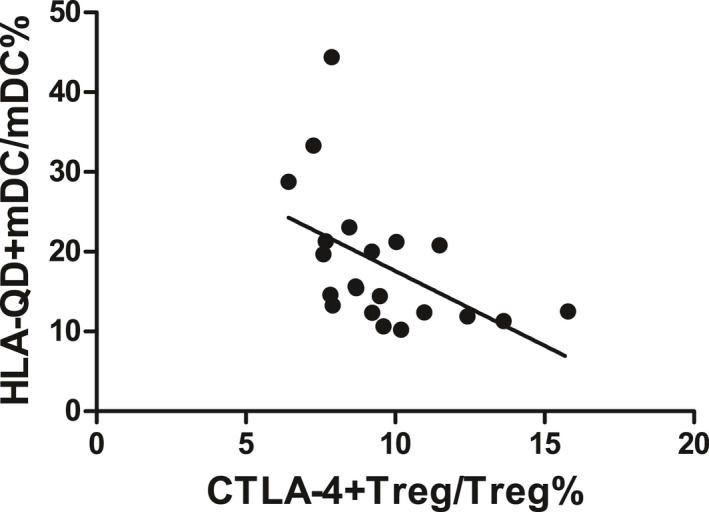
Negative correlation between the CTLA‐4 expression on Tregs and the HLA‐DQ expression on mDCs in SAA patients

There was a negative correlation between CTLA‐4 expression on Tregs and HLA‐DQ expression on mDCs in SAA patients (*r* = −.500, *P* < .05, Figure 4).

### 3.4 Correlations of the CTLA‐4 expression on Tregs and the HLA‐DQ expression on mDCs with the numbers of other immune cells in SAA patients

**FIGURE 5:**
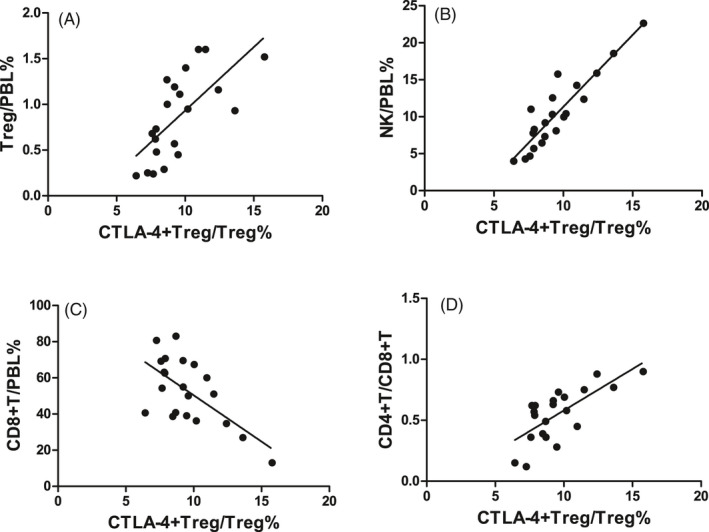
Correlations between CTLA‐4 expression on Tregs and the numbers of other immune cells in SAA patients. A, CTLA‐4^+^Treg/Treg% and Treg/PBL% were positively correlated. B, CTLA‐4^+^Treg/Treg% and NK/PBL% were positively correlated. C, CTLA‐4^+^Treg/Treg% and CD8^+^T/PBL% were negatively correlated. D, CTLA‐4^+^Treg/Treg% was positively correlated with the CD4^+^T/CD8^+^T ratio

In the SAA patients, CTLA‐4 expression on Tregs was positively correlated with Treg/PBL (*r* = .683, *P* < .01, Figure 5A), NK/PBL (*r* = .856, *P* < .001, Figure 5B), and the CD4^+^T/CD8^+^T ratio in PB (*r* = .700, *P* < .001, Figure 5D) but negatively correlated with CD8^+^T/PBL (*r* = −.540, *P* < .05, Figure 5C).

**FIGURE 6:**
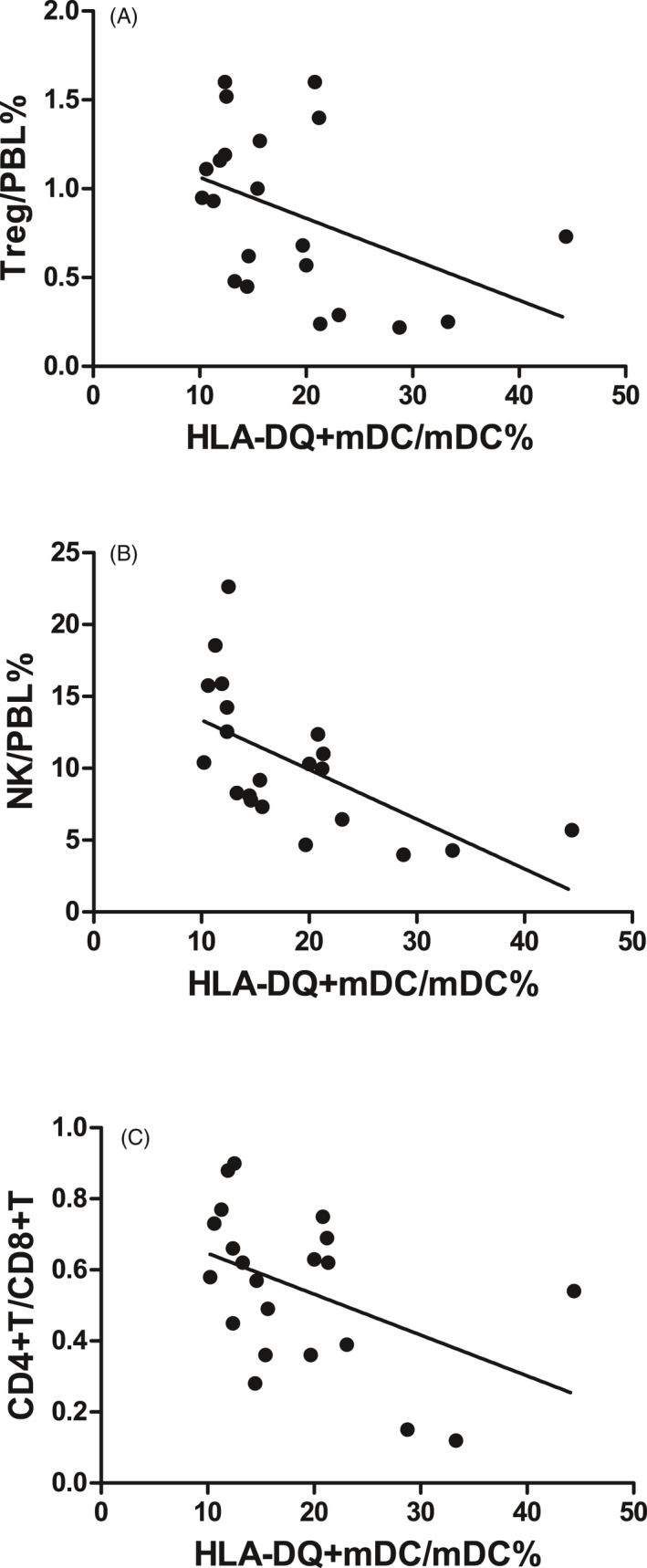
Correlations between HLA‐DQ expression on mDCs and the numbers of other immune cells in SAA patients. A, HLA‐DQ^+^mDC/mDC% and Treg/PBL% were negatively correlated. B, HLA‐DQ^+^mDC/mDC% and NK/PBL% were negatively correlated. C, HLA‐DQ^+^mDC/mDC% and the CD4^+^T/CD8^+^T ratio were negatively correlated

In the SAA patients, HLA‐DQ expression on mDCs was negatively correlated with Treg/PBL (*r* = −.450, *P* < .05, Figure 6A), NK/PBL (*r* = −.540, *P* < .05, Figure 6B), and the CD4^+^T/CD8^+^T ratio in PB (*r* = −.487, *P* < .05, Figure 6C) but was not correlated with CD8^+^T/PBL (*r* = .403, *P* = .070).

### 3.5 Correlations of CTLA‐4 expression on Tregs and HLA‐DQ expression on mDCs with clinical disease states in SAA patients

**FIGURE 7:**
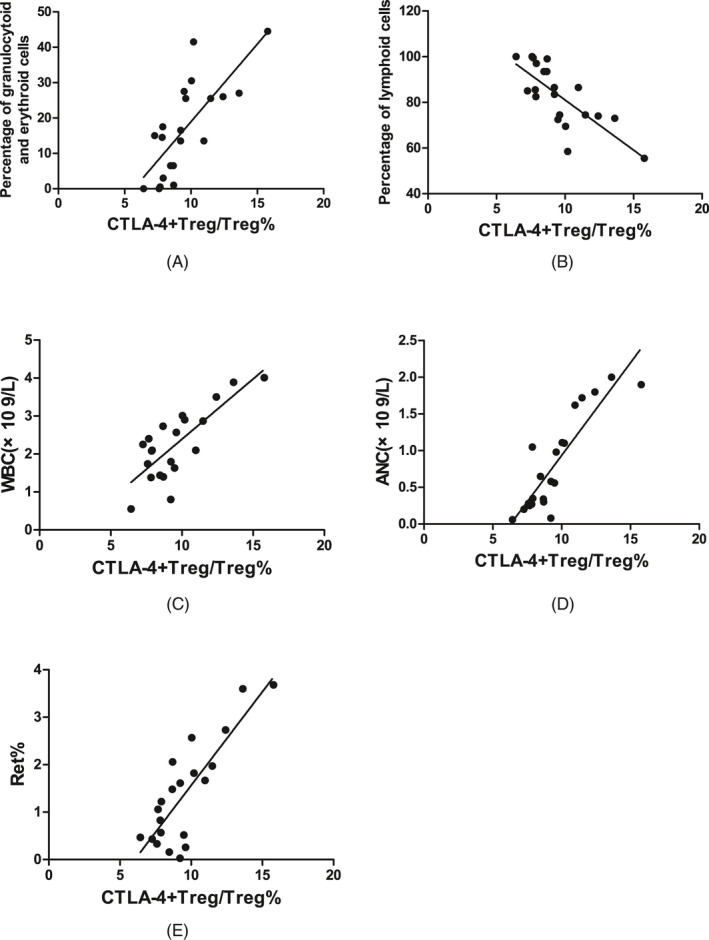
Correlations between CTLA‐4 expression on Tregs and the relevant indicators of clinical disease status in SAA patients. A, CTLA‐4^+^Treg/Treg% was positively correlated with the percentage of granulocytoid and erythroid cells in bone marrow. B, CTLA‐4^+^Treg/Treg% was negatively correlated with the percentage of lymphoid cells in bone marrow. C, CTLA‐4^+^Treg/Treg% was positively correlated with WBC count in PB. D, CTLA‐4^+^Treg/Treg% was positively correlated with ANC in PB. E, CTLA‐4^+^Treg/Treg% was positively correlated with the percentage of Rets in PB

In SAA patients, the CTLA‐4 expression on Tregs was positively correlated with the percentage of granulocytoid and erythroid cells in bone marrow (*r* = .752, *P* < .001, Figure 7A), the WBC count in PB (*r* = .672, *P* < .01, Figure 7C), ANC in PB (*r* = .870, *P* < .001, Figure 7D), and the percentage of Rets in the PB (*r* = .687, *P* < .01, Figure 7E); was negatively correlated with the percentage of lymphoid cells in bone marrow (*r* = −.757, *P* < .001, Figure 7B); and was not statistically correlated with the megakaryocyte number in bone marrow, absolute PBL count, HGB in PB, absolute Ret count in PB, or PLT count in PB.

**FIGURE 8:**
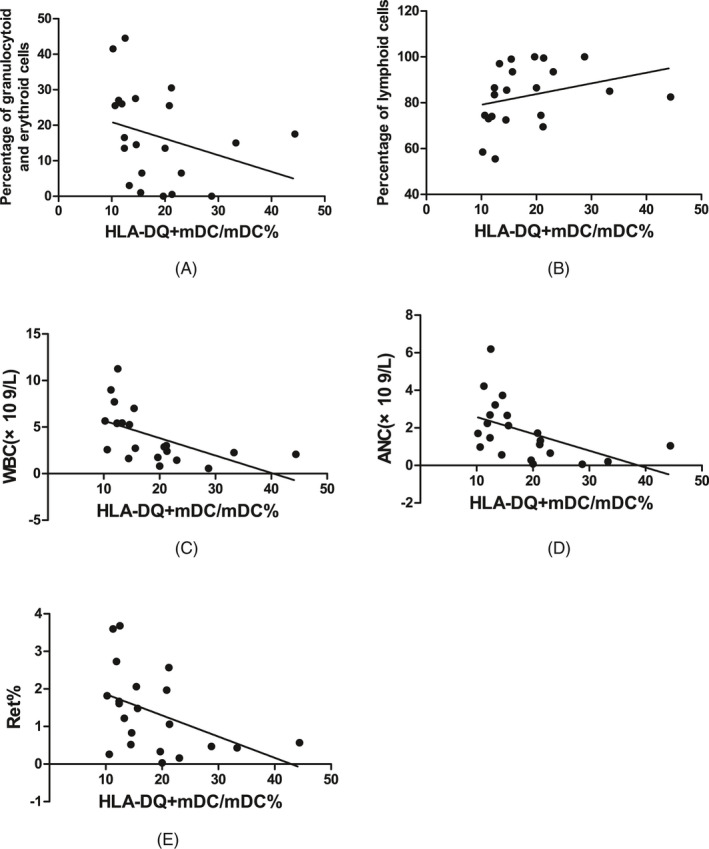
Correlations between HLA‐DQ expression on mDCs and the relevant indicators of clinical disease status in SAA patients. A, HLA‐DQ^+^mDC/mDC% was negatively correlated with the percentage of granulocytoid and erythroid cells in bone marrow. B, HLA‐DQ^+^mDC/mDC% was positively correlated with the percentage of lymphoid cells in bone marrow. C, HLA‐DQ^+^mDC/mDC% was negatively correlated with WBC count in PB. D, HLA‐DQ^+^mDC/mDC% was negatively correlated with the ANC in PB. E, HLA‐DQ^+^mDC/mDC% was negatively correlated with the percentage of Rets in PB

In SAA patients, the HLA‐DQ expression on mDCs was negatively correlated with the percentage of granulocytoid and erythroid cells in bone marrow (*r* = −.449, *P* < .05, Figure 8A), WBC count in PB (*r* = −.555, *P* < .01, Figure 8C), ANC in PB (*r* = −.491, *P* < .05, Figure 8D), and the percentage of Rets in PB (*r* = −.441, *P* < .05, Figure 8E); was positively correlated with the percentage of lymphoid cells in bone marrow (*r* = .449, *P* < .05, Figure 8B); and was not statistically correlated with the megakaryocyte number in bone marrow, absolute PBL count, HGB in PB, absolute Ret count in PB, or PLT count in PB.

### 3.6 Changes in the number of Tregs, CTLA‐4 expression on Tregs, HLA‐DQ expression on mDCs, NK cell number, CD8^+^ T‐cell number, and CD4^+^T/CD8^+^T ratio in SAA patients after IST

To study the changes in the number of Tregs, CTLA‐4 expression on Tregs, HLA‐DQ expression on mDCs, NK cell number, CD8^+^ T‐cell number, and CD4^+^T/CD8^+^T ratio in SAA patients from before to after enhanced IST, 10 patients in the SAA group, whose IST with rATG and CsA was effective, were observed before and at 3 and 6 months after IST.

**FIGURE 9:**
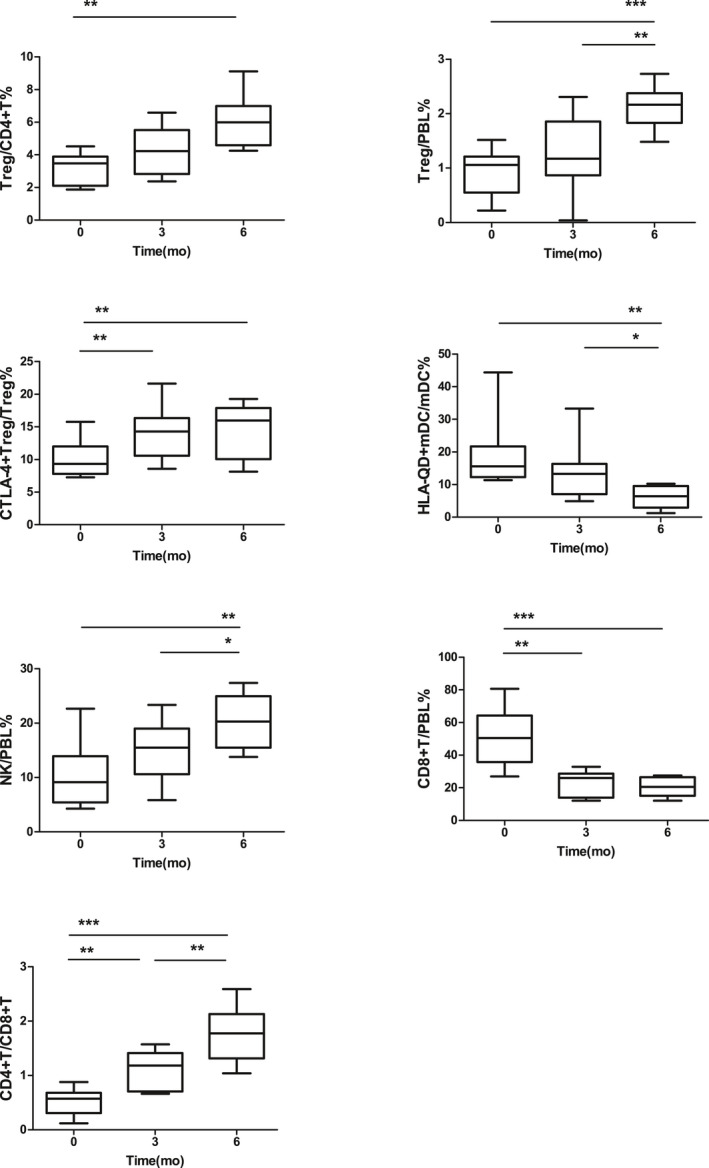
Changes in the number of Tregs, the CTLA‐4 expression on Tregs, the HLA‐DQ expression on mDCs, the NK cell number, the CD8^+^ T‐cell number, and the CD4^+^T/CD8^+^T ratio in SAA patients from before to after IST; ***P < .001, **P < .01, *P < .05

In these SAA patients, Treg/CD4^+^T in the PB was 3.24 ± 0.96%, 4.25 ± 1.42%, and 6.02 ± 1.51% before and at 3 and 6 months after IST, respectively. The Treg/CD4^+^T at 6 months after IST was significantly greater than before treatment (*P* < .01). There was no significant difference between Treg/CD4^+^T at 3 months after IST and that before IST (Figure 9). Treg/PBL in the PB was 0.91 ± 0.42%, 1.29 ± 0.70%, and 2.12 ± 0.38% before and 3 and 6 months after IST, respectively. Treg/PBL at 6 months after IST was significantly greater than before IST (*P* < .001), though there was no significant difference between Treg/PBL at 3 months after IST and that before IST (Figure 9). CTLA‐4^+^Treg/Treg was 10.08 ± 2.78%, 14.32 ± 4.15%, and 14.34 ± 4.33% before and 3 and 6 months after IST, respectively. CTLA‐4^+^Treg/Treg at 3 and 6 months after IST was significantly higher than before IST (*P* < .05, *P* < .05), and the difference between 3 and 6 months after IST was not statistically significant (Figure 9). HLA‐DQ^+^mDC/mDC was 18.77 ± 9.94%, 13.75 ± 8.25%, and 6.12 ± 3.40% before and 3 and 6 months after IST, respectively. HLA‐DQ^+^mDC/mDC at 6 months after IST was significantly lower than before and 3 months after IST (*P* < .01, *P* < .05), and there was no significant difference in HLA‐DQ^+^mDC/mDC between 3 months after IST and before IST (Figure 9). NK/PBL in the PB was 10.50 ± 5.98%, 14.69 ± 5.41%, and 20.41 ± 5.08% before and 3 and 6 months after IST, respectively. NK/PBL at 6 months after IST was significantly higher than before IST (*P* < .01), though no statistically significant difference was found in NK/PBL between 3 months after IST and before IST (Figure 9). CD8^+^T/PBL in the PB was 51.06 ± 17.12%, 23.25 ± 7.52%, and 20.48 ± 5.58% before and 3 and 6 months after IST, respectively. CD8^+^T/PBL at 3 and 6 months after IST was significantly lower than before IST (*P* < .01, *P* < .001), and the difference between 3 and 6 months after IST was not statistically significant (Figure 9). The CD4^+^T/CD8^+^T ratio was 0.52 ± 0.24%, 1.09 ± 0.36%, and 1.76 ± 0.50% before and 3 and 6 months after IST, respectively. The CD4^+^T/CD8^+^T ratio at 3 and 6 months after IST was significantly lower than before IST (*P* < .01, *P* < .001), and the CD4^+^T/CD8^+^T ratio at 6 months was also significantly higher than that at 3 months (*P* < .01, Figure 9).

## 4 DISCUSSION

Severe aplastic anemia is a severe hematologic disease characterized by a severe reduction in whole blood cells and bone marrow failure. Its pathogenesis is directly related to the damage done to autologous bone marrow hematopoietic cells by T‐cell immune dysfunction. In clinical practice, the well‐known manifestations of SAA are that the number of CD8^+^ T cells increases and the CD4^+^T/CD8^+^T ratio is inverted and significantly reduced. According to previous studies, the immune pathogenesis of SAA is that the mDCs are activated by some unknown antigens, express major histocompatibility complex (MHC) class II molecules, present antigens to help T cells (Th), induce the differentiation of Th0 to Th1, cause a Th1/Th2 imbalance, produce many type I lymphokines, and activate the CD8^+^ T cells, thus resulting in bone marrow failure. In addition, the NK cell number decreases during the whole process of immune activation, and NK cells seem to have an immunoregulatory function to limit the maturation and excessive activation of DCs.^10^

Tregs are a T lymphocyte subset with immunoregulatory functions. Tregs play an inhibitory role in the autoimmune response and have the function of maintaining immune tolerance status. The immunoregulation of Tregs mainly inhibits T‐cell proliferation and the production of cytokines and autoantibodies and regulates the functions of DCs, mononuclear macrophages, and NK cells. Previous studies have suggested that the number of Treg cells decreases in SAA patients and gradually recovers after IST.^11^ Solomou et al^12^ studied the Tregs in SAA patients and showed that the expression levels of the *FOXP3* gene and its encoded protein scurfin were decreased in CD4^+^CD25^+^ cells, and the activation of nuclear factor of activated T cells (NFAT) 1 was insufficient or even absent. Scurfin controls NFAT expression to induce the production and functioning of Tregs. FoxP3 gene mutations in human and mouse can cause severe autoimmune diseases in multiple organs. In the CD4^+^CD25^+^FoxP3^‐^ cells of AA patients transfected with NFAT1, the expression of NFAT1 is increased, and the expression of FoxP3 is also increased. In contrast, when NFAT1 is knocked out from Tregs, Foxp3, and NFAT1 expression is decreased, suggesting that the cells that induce immune tolerance are seriously insufficient in SAA patients.

During the immune response, DCs recognize, take in, and process antigens; synthesize MHC‐antigen (peptide) complexes and costimulatory molecules on the surface; and present antigens to naïve T cells. This process plays an important role in the immune response. Our previous results suggested that the number of mDCs in SAA was increased, the mDC/pDC ratio was increased, and the mDCs were hyperfunctional.^13^ The abnormal antigen‐presenting process of mDCs is an important step in SAA immune activation, which may be the starting point of SAA immune cascade activation. In addition, MHC‐II molecules mainly perform the function of presenting antigens to CD4^+^ T cells and are the most critical proteins in the antigen‐presenting process of mDCs.^14^ Tregs damage the DCs through perforin and granzymes^15^ and can synthesize the immunosuppressive molecule adenosine, which binds to the adenosine receptor on the surface of DCs and inhibits the expression of intracellular inflammatory cytokines, thereby inhibiting the activation and proliferation of DCs.^16^ Tregs can also inhibit the functions of the above immune cells through direct contact.^17^ DCs are the main regulatory target of Tregs, and this regulatory process relies on antigenic stimulation.^18^

This study analyzed the regulatory mechanism of Tregs on mDCs. The experimental results suggested that the number of Tregs in the PB of SAA patients was decreased significantly and gradually recovered after IST. CTLA‐4 expression on Tregs was also significantly reduced and was restored after enhanced IST. CTLA‐4 expression was positively correlated with the number of Tregs. These results suggest that the reduced number and decreased inhibitory effect of Tregs were the reasons for the disruption of the immune tolerance in SAA, and CTLA‐4 was the key molecule in this process.

CTLA‐4 is an important inhibitor of T‐cell function. After immune activation, CTLA‐4 is abundantly expressed on Tregs to transduce inhibitory signals and maintain immune response homeostasis.^19^ The deletion of the CTLA‐4 haplotype can cause autoimmune diseases, multiorgan invasion by lymphocytes, and the peripheral B cells and immunoglobulin deficiency.^20^, ^21^ After the CTLA‐4 gene is knocked out or the protein is neutralized by antibodies, Tregs cannot normally perform their immunosuppressive functions, leading to abnormal immune regulation.^19^ Single‐nucleotide polymorphisms in CTLA‐4 have been correlated with a variety of autoimmune diseases, such as rheumatoid arthritis, systemic lupus erythematosus, inflammatory bowel disease, type 2 diabetes, and rheumatoid arthritis.^22^, ^23^, ^24^, ^25^ In the treatment of tumors using CTLA‐4 monoclonal antibody (ipilimumab), the patients showed significant upregulation of the Th1 and Th17 pathways and adverse reactions of inflammatory bowel disease.^26^, ^27^ We also examined the expression of MHC class II molecule HLA‐DQ on mDCs in SAA patients, which was significantly higher than that in HCs and was decreased after IST. Correlation analysis revealed a negative correlation between CTLA‐4 expression on Tregs and HLA‐DQ expression on mDCs. Next, the correlations of the CTLA‐4 expression on Tregs and HLA‐DQ expression on mDCs with the numbers of other immune cells were analyzed. The results showed that in SAA patients, the CTLA‐4 expression on Tregs was positively correlated with the NK cell number and CD4^+^T/CD8^+^T ratio in PB and was negatively correlated with the CD8^+^ T‐cell number in PB; the HLA‐DQ expression on mDCs was negatively correlated with the number of Tregs, NK cell number, and CD4^+^T/CD8^+^T ratio in PB. These results suggested that in SAA patients, the number of Tregs and the CTLA‐4 expression were decreased, and abnormal activation of mDCs may have been caused by the insufficient regulation of the antigen‐presenting process of mDC MHC class II molecules, initiating a series of immune cell activation processes. Moreover, the regulatory effect of Tregs on mDCs may also be jointly realized by two key molecules, CTLA‐4 and HLA‐DQ. A recent study on renal transplant rejection showed that binding of HLA‐DQ antibodies to endothelial cells could selectively reduce T‐cell proliferation and the differentiation of FoxP3^high^ Tregs. After endothelial cells are stimulated by HLA‐DQ antibodies, a synergistic increase in pro‐inflammatory cytokine secretion and reduction in Treg amplification was observed,^28^ suggesting that MHC class II molecules can also inhibit Tregs. Future studies on MHC class II molecules that impair immune tolerance may provide ideas for searching for antigenic substances that stimulate the pathogenesis of SAA.

The correlations of the CTLA‐4 expression on Tregs and HLA‐DQ expression on mDCs with the clinical hematopoietic parameters were analyzed. The results showed that in SAA patients, the CTLA‐4 expression on Tregs was positively correlated with the percentage of granulocytoid and erythroid cells in bone marrow, the WBC count in PB, ANC in PB, and the percentage of Rets in PB but was negatively correlated with the percentage of lymphoid cells in bone marrow. The HLA‐DQ expression on mDCs was negatively correlated with the percentage of granulocytoid and erythroid cells in bone marrow, WBC count in PB, ANC in PB, and the percentage of Rets in PB but was positively correlated with the percentage of lymphoid cells in bone marrow. Analyses of the changes in expression levels of CTLA‐4 on Tregs and HLA‐DQ on mDCs and the numbers of various immune cells before and after IST showed that in SAA patients, the number of Tregs in PB, the HLA‐DQ expression on mDCs, and the NK cell number at 3 months after IST were not different from those before IST, but they were significantly higher 6 months after IST than before IST; the CTLA‐4 expression on Tregs and CD8^+^ T‐cell number at 3 months after IST was significantly higher than before IST, and those at 3 months after IST were not significantly different from those at 6 months after IST; the CD4^+^T/CD8^+^T ratio was significantly higher at 3 months after IST than before IST and continued to increase at 6 months after IST. In SAA patients, CTLA‐4 expression on Tregs and HLA‐DQ expression on mDCs was not statistically correlated with the megakaryocyte number in bone marrow, absolute PBL count, HGB in PB, absolute Ret count in PB, or PLT count in PB. The reasons were as follows: (1) The detected megakaryocyte number in bone marrow in the SAA patients was almost zero, so no correlation was found. (2) The acute SAA had a short disease course, and the neutrophils and reticulocytes decreased rapidly after many hematopoietic cells were killed, but the lifespan of mature red blood cells in PB is long, so some patients did not show significant declines. (3) Patients may receive frequent infusion of red blood cells, platelets, and other blood components at the beginning of the disease, resulting in the inability to accurately determine the real levels at the onset. (4) The SAA patients had a severely low blood cell count, leading to the proportions of lymphocytes and reticulocytes out of nucleated cells but not the absolute values could accurately reflect the correlation. Changes in various immune indicators from before to after IST showed that the number of Tregs recovered at 6 months after IST, and CTLA‐4 expression on Tregs started to recover at 3 months after IST, suggesting that CTLA‐4 expression on Tregs may be sensitive and active in the activation and recovery of SAA immunity and may play an important role in the recovery of the number and function of Tregs and even the recovery of entire immune status.

CTLA‐4 monoclonal antibody has achieved clear efficacy in the treatment of tumors, and it is used as a therapeutic target in research on SAA and other autoimmune diseases.^29^ The CD8^+^ T‐cell number and CD4^+^T/CD8^+^T ratio were significantly corrected at 3 months after IST and continued to recover over time. The NK cell number started to recover at 6 months after IST, which was similar to the recovery of the number of Tregs, suggesting that the inhibitory factors in the SAA immunization cascade recover less readily. MHC class II HLA‐DQ on mDCs also showed this pattern after IST, which suggested that it may be a target in Tregs regulating mDCs, providing an idea for research on the poor efficacy of IST and on the recurrence monitoring of diseases.

In summary, the number of Tregs and their functional molecule CTLA‐4 were significantly downregulated in SAA, and the expression of the MHC class II molecule HLA‐DQ on mDCs was significantly upregulated. These changes might be key to the loss of regulation by Tregs of mDCs in SAA patients. These findings are conducive to further exploring the mechanism of SAA immune pathogenesis. Research on the key regulatory mechanisms of Tregs and CTLA‐4 has great potential to help find therapeutic targets of SAA and to monitor this disease.

## CONFLICT OF INTEREST

The authors declare no conflicts of interest.
